# Removal of formaldehyde from indoor air by potted *Sansevieria trifasciata* plants: dynamic influence of physiological traits on the process

**DOI:** 10.1007/s11356-024-35366-4

**Published:** 2024-10-29

**Authors:** Jian Li, Silan Chen, Jiaochan Zhong, Shujie Lin, Shifan Pang, Qianying Tu, Igor Agranovski

**Affiliations:** 1 School of Environmental and Chemical Engineering, Nanchang Hangkong University, Nanchang, 330063 China; 2 School of Engineering and Built Environment, Griffith University, Brisbane, QLD 4111 Australia

**Keywords:** Formaldehyde stress, Dynamic fumigation, Phytohormone, Antioxidant oxidase, Plant growth-promoting endophytes

## Abstract

Plant-based removal of indoor formaldehyde is a widely studied method, yet little is known about the dynamic changes in this process. In this study, potted *Sansevieria trifasciata* Prain plants were exposed to 5-ppm formaldehyde gas concentration for 7 days. The results showed that formaldehyde exposure led to plant stress, affected photosynthesis, and damaged membrane lipids, as evidenced by a decrease in chlorophyll content, an increase in Chl a/b ratio and malondialdehyde content. However, the formaldehyde removal ability of the plants increased over the first 5 days, peaking at 18.02 mg h^−1^ kg^−1^ dry weight on the 5th day. This trend was correlated with changes in various indicators in the plant roots, including phytohormone and antioxidant enzymes. Notably, catalase activity in the roots behaved differently from other indicators. The indicators in the leaves showed turning points around the 3rd day due to the direct exposure of the leaves to formaldehyde. The relative abundance of endophytes indicated an increase in plant growth-promoting bacteria, which helped the plant cope with formaldehyde stress. The study suggests that under formaldehyde stress, plants manage active oxygen content by increasing phytohormones and regulating redox reactions. This enhances their tolerances to formaldehyde, thereby improving their ability to remove formaldehyde and aiding recovery after formaldehyde exposure.

**Graphical abstract:**

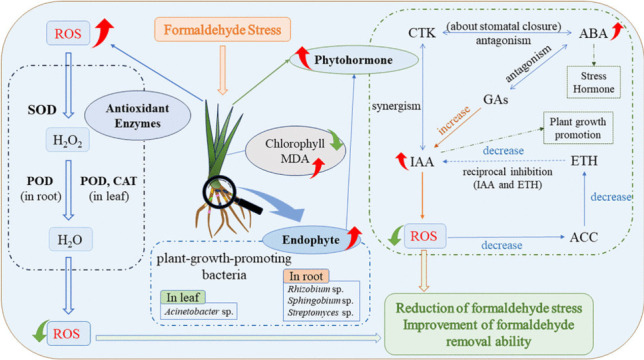

**Supplementary Information:**

The online version contains supplementary material available at 10.1007/s11356-024-35366-4.

## Introduction

Formaldehyde is classified as a group 1 carcinogen by the International Agency for Research on Cancer (IARC) (Shao et al. 2020). The emission of formaldehyde from indoor building materials is a gradual process, so the concentration of formaldehyde indoors is higher than outdoors (Salthammer et al. 2010). Most people spend 95% of their time indoors. Prolonged exposure to indoor pollutants, including formaldehyde, can lead to sick building syndrome (SBS) and building-related illness (BRI) (Kelly and Fussell 2019). Consequently, there is a growing concern about the need to mitigate indoor formaldehyde levels for the well-being of occupants. The World Health Organization recommends that indoor concentrations of formaldehyde should be less than 0.1 mg m^−3^ for 30 min (World Health Organization 2000).

Various methods are employed for indoor formaldehyde removal, encompassing physical (ventilation, adsorption), chemical (catalytic oxidation), and biological (plant and microbial removal) approaches. Although simple, opening windows for ventilation is a slow process and can lead to heat loss in winter, presenting challenges in regions with additional air pollution (Miao et al. 2019). Adsorption on porous materials is effective but comes with high costs for adsorbents and necessitates regular replacement or regeneration (Luengas et al. 2015). Catalytic oxidation demands strict conditions and expensive catalysts, some of which can be harmful to humans, potentially introducing secondary pollutants (Shao et al. 2020). Biological methods especially for plant degradation have gained attention due to their safety, environmental friendliness, and the absence of secondary pollution (Khalifa et al. 2023). These methods leverage biological activities to assimilate formaldehyde into biological components or dissimilate it into substances such as CO_2_, releasing it into the external environment.

The method of employing plants for air pollutant removal has emerged as a crucial choice due to its cost-effectiveness, straightforward operation, and ready availability (Teiri et al. 2018). Numerous studies have demonstrated the significant efficacy of various plants in formaldehyde removal, including *Chlorophytum comosum* (Thunb.) Jacques (Li et al. 2019; Siswanto et al. 2020), *Epipremnum aureum* (Linden & André) Bunting (Xu et al. 2011), *Aloe vera* (L.) Burm. f. (Xu et al. 2011), *Sansevieria trifasciata* Prain (Zhao et al. 2019; Siswanto et al. 2020), *Chamaedorea elegans* Mart. (Teiri et al. 2018), *Lycopersicon esculentum* L. (Zhao et al. 2019), and *Helianthus annuus* L. (Zhao et al. 2019). Among these plants, most are classified as C3 plants, utilizing the C3 photosynthetic carbon reduction cycle. In contrast, *S. trifasciata* and *A. vera* follow caryophyllotoxic acid metabolism (CAM). Beyond formaldehyde, plants also help remove other indoor air pollutants. For instance, *Euphorbia milii* Des Moul. demonstrated removal efficiencies of 70.23% for 20 ppm benzene in 96 h, 100.00% for 20 ppm formaldehyde in 48 h, and 90.32% for 100 ppm trimethylamine in 16 h (Khaksar et al. 2016a). Specifically, the formaldehyde removal rate of *S. trifasciata* is 11.31 ± 0.10 µg h^−1^ g^−1^ FW, and the formaldehyde concentration within 24 h is 0.56 ± 0.04 mg m^−3^ (Zhao et al. 2019). In addition, *S. trifasciata* plants exhibit high tolerance to volatile organic compounds (VOCs). This enhances the plant’s resilience to VOC stress by boosting metabolic processes like photosynthesis and redox balance (Permana et al. 2023). Additionally, endophytic microorganisms residing in plant tissues significantly contribute to VOC removal. They offer various benefits to host plants, including enhanced nutrient availability, production of plant growth hormones, and control of soil pathogens, thereby fostering overall plant growth (Khaksar et al. 2016a).

While numerous studies have delved into the mechanisms and efficiency of formaldehyde removal by plants, there is a noticeable scarcity of literature detailing the associated alterations in biological parameters of plants during the removal processes. Compared with other plants mentioned, although *S. trifasciata* does not have the best formaldehyde removal ability, it is highly adaptable, has a strong tolerance to VOCs, and is non-toxic to humans. As a CAM plant, *S. trifasciata* is more adaptable to unfavorable survival conditions such as drought and high temperature. In contrast, plants of the family Amanita, including *E. aureum* and *M. deliciosa*, are toxic, although they have a better effect on formaldehyde removal, and therefore were not considered as experimental subjects. This study addresses this gap by investigating the dynamic processes of formaldehyde fumigation on *S. trifasciata* plants. The objectives include assessing the removal ability, analyzing the stress response of both roots and leaves, and examining changes in endophytes during the testing process.

## Experimental design

### Reagents and plant materials

The reagents used in this study were purchased from the National Medicines Corporation Ltd. (Shanghai, China), including HCHO (40%), KH_2_PO_4_ (99.5%), K_2_HPO_4_ (99.0%), NaCl (99.5%), NaH_2_PO_4_·2H_2_O (99.0%), Na_2_HPO_4_·12H_2_O (99.0%), C_2_H_5_OH (99.7%), and NaClO (8%). All reagents and calibration standards adhered to analytical grade chemicals. *S. trifasciata* plants were bought from Nanchang flower market and grown in pots (10 cm × 12 cm, height × diameter) filled with 0.2 kg of soil, consisting of 1:1 garden soil and humus soil (v/v). Under vendor suggested conditions in laboratory, the potted plants were watered every 5 days during the period of plant domestication, kept in a ventilated environment, and grown under natural light for 1 month.

### Fumigation experiment

**Fig. 1:**
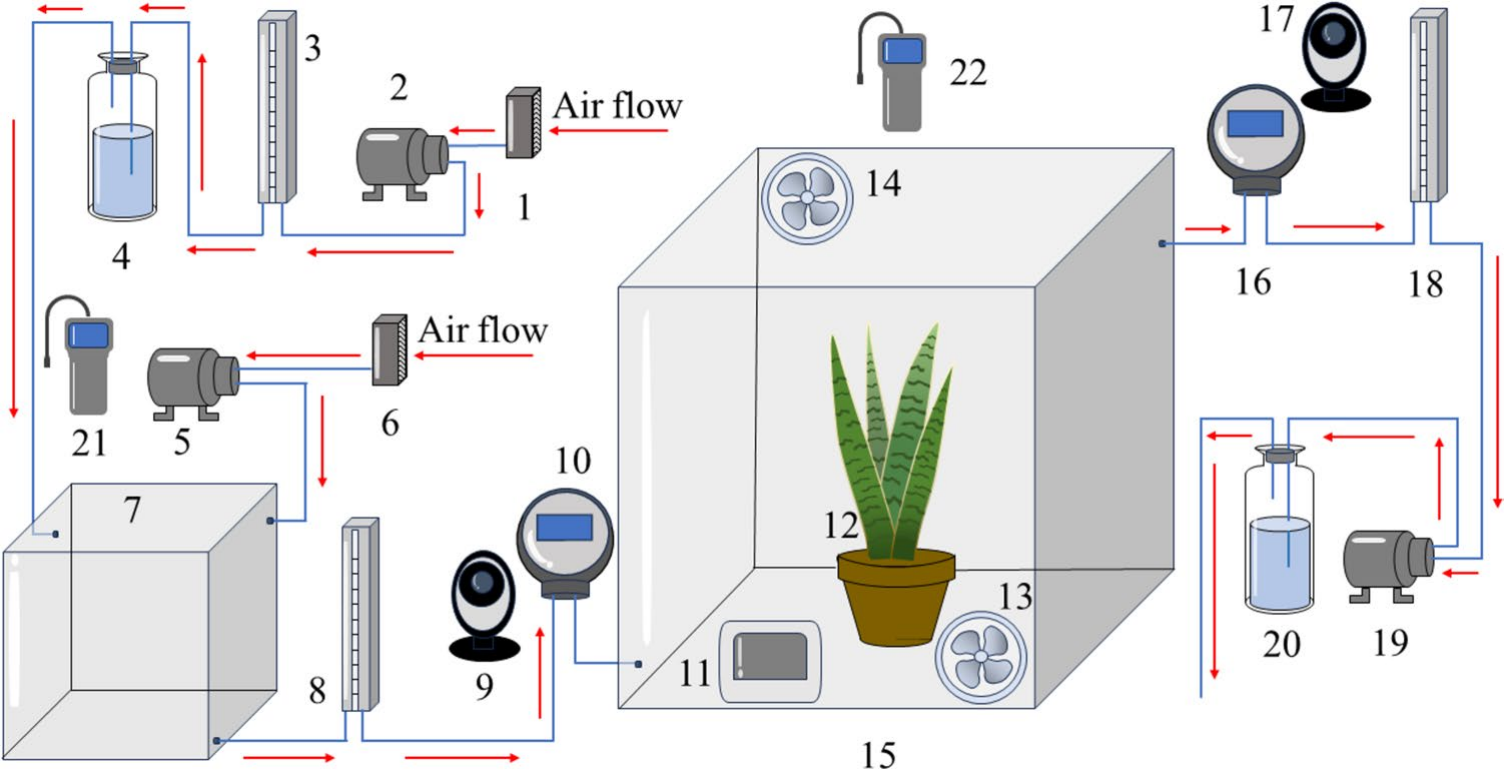
Fumigation apparatus (1, 6—HEPA filter; 2, 5, 19—air pump; 3, 8, 18—rotameter; 4—formaldehyde solution; 7—gas mixing chamber; 9, 17—camera; 10, 16—formaldehyde detector; 11—hygrothermoscope; 12—test potted plant; 13, 14—small fan; 15—fumigation chamber; 20—2,4-dinitrophenylhydrazine (DNPH) hydrochloric acid solution; 21, 22—leak detector)

A laboratory setup, shown in Fig. 1 (also, the photograph is available in Supplementary materials 1), was used for fumigation experiments on potted *S. trifasciata* plants. Formaldehyde gas was produced by bubbling HEPA filtered air (1) through formaldehyde solution (4) by the air pump (2) (HLVP8-WB24-S, Kamoer, Shanghai, China). To control the concentration of formaldehyde in the experimental lines, an additional make-up flow of HEPA-filtered air was supplied to the gas mixing chamber (7) by a pump (5), mixed with formaldehyde gas, and transported to the 0.125 m^3^ (length × width × height = 0.5 × 0.5 × 0.5 m) test chamber (15) to expose plants (12) to formaldehyde. The air flow rate was monitored and controlled by the rotameter (3) to determine the formaldehyde concentration at the inlet at a level of 5 ± 0.5 ppm. The pots of the plants were tightly wrapped with tin foil ensuring only ground part of the plant exposed to formaldehyde. The temperature was continuously measured by a thermohydrometer (TH1018, Hegao, Guangzhou, China) (11). The gas in the test chamber was transported by another air pump (19) and passed through a hydrochloric solution of 2,4-dinitrophenylhydrazine (DNPH) in a clean vessel (20) to capture the exhausted formaldehyde gas in the system. Inlet and outlet formaldehyde concentrations were monitored using on-line formaldehyde detectors (ADL-600B-HCHO, ANDEIL, Shenzhen, China, measurement range/resolution: 0–10 ppm/0.01 ppm; sensor type: relay, 1 set (1A/24VDC); measurement accuracy/resolution: ≤  ± 3%/ ≤  ± 1% (F.S/year)) (10, 16) and recorded in real time by two network cameras (1080P, YI, Shanghai, China) (9, 17). The flow rate of inlet and outlet gas was controlled at 120 L h^−1^ by rotameter (LZB-4, Dongxing, China) (8, 18).

To investigate the formaldehyde removal ability, *S. trifasciata* plants with similar height, weight, leaf width, and other growth conditions (refer to Table 1) were meticulously chosen for the experiment. These selected plants underwent fumigation for 0, 1, 3, 5, and 7 days. Simultaneously, a control group was established and subjected to clean air, while a blank group was placed in a standard indoor environment outside the test chamber for the entire 7-day period. Throughout the experiment, the average temperature and humidity inside and outside the chamber were monitored. The conditions were as follows: average temperatures outside (inside) the chamber were 27.1 ℃ (26.4 ℃), ranging from 23.3 ℃ (23.1 ℃) to 29.4 ℃ (29.1 ℃). Humidity levels ranged from 69.5% (72.3%) to 90.5% (87.6%), respectively. During the experiment, the light intensity was 316–871 Lux (daytime) and 0–65.7 Lux (nighttime).

**Table 1:** Plant characteristics of *S. trifasciata* for fumigation experiments

Height (cm)	Dry weight (g)	Leaf width (cm)	Number of leaves	Formaldehyde fumigation time (d)
31.32 ± 1.33	8.36 ± 0.33	6.13 ± 0.83	4	0
31.51 ± 0.89	8.42 ± 0.36	6.51 ± 1.04	4	1
31.45 ± 1.16	8.39 ± 0.28	7.02 ± 0.47	4	3
31.53 ± 1.18	8.44 ± 0.41	6.78 ± 0.66	4	5
31.46 ± 1.13	8.37 ± 0.24	6.34 ± 1.12	4	7
31.55 ± 1.27	8.38 ± 0.39	6.37 ± 0.54	4	Control
31.78 ± 1.19	8.41 ± 0.28	6.64 ± 0.92	4	Blank

### Leak test

A leak test on the entire equipment was conducted before starting the experiments. The primary goal was to verify the airtightness of the fumigation system, and the secondary objective was to assess the extent of formaldehyde adsorption by the inner walls of the equipment. Before conducting the leak test, the average background formaldehyde concentration in the laboratory was measured over a 24-h period without operating the fumigation apparatus. This was done using a leak detector (WP6912, AGERUISI, Beijing, China) with a measurement range of 0.000–1.999 mg m^-^^3^ and a resolution of 0.001 mg m^-^^3^. The concentration was measured at 54.9 ± 9.95 µg/m3 (0.045 ± 0.0075 ppm), which is significantly lower than the permissible exposure limit (PEL) for formaldehyde in the workplace, set at 0.75 ppm as an 8-h time-weighted average, according to the recommendations of the Occupational Safety and Health Administration (OSHA 1990).

Then, for the leak test, formaldehyde gas with a concentration of 5 ± 0.5 ppm was continuously introduced into the experimental apparatus at a flow rate of 1.2 L min^-1^ for 24 h. It was found that the inflow concentration of formaldehyde remained stable at 5.05 ± 0.10 ppm, while the outflow concentration was maintained at 4.99 ± 0.12 ppm. The system achieved an average gas recovery rate of 98.73%, demonstrating effective sealing and confirming its suitability for proceeding with further experiments. The formaldehyde concentration around the apparatus ranged between 40 and 65 μg m^-^^3^ (0.032 to 0.053 ppm), confirming that there was no leakage from the equipment to the laboratory environment, as the concentration did not exceed the background levels. To further elaborate, the measured formaldehyde concentration levels surrounding the apparatus suggested effective containment and proper sealing of the equipment during operation. These results confirm that the fumigation system is functioning as intended, without any detectable leakage, thereby ensuring safety and compliance with health standards. Consistent monitoring of these levels is crucial to maintain a safe working environment. All details of the procedure along with the raw data on the leak test are available in the Supplementary materials 2.

### Determination of physiological and biochemical indexes of plants

#### Determination of chlorophyll content

The chlorophyll content in *S. trifasciata* leaves was assessed through spectrophotometry, following the method outlined by Li et al. (2019). Fresh leaves (0.5 g) of *S. trifasciata* were collected, ground into a tissue homogenate, and combined with a 10 mL mixed solution of acetongee and anhydrous ethanol (1:1, v/v). The mixture was left in a dark environment until the leaves’ tissue turned white. Subsequently, the absorbance of the extract was measured using a UV–VIS spectrophotometer (UV-5200, METASH, Shanghai, China) at wavelengths of 645 nm and 663 nm. The chlorophyll content was determined using the following formulas (1) to (3):

1 $${\text{W}}_{\text{a}}=\left(12.72\times \text{A}663-2.59\times \text{A}645\right)\times \text{v}/\left(1000\times \text{m}\right)$$

2 $${\text{W}}_{\text{b}}=\left(22.88\times \text{A}645-4.67\times \text{A}663\right)\times \text{v}/\left(1000\times \text{m}\right)$$

3 $${\text{W}}_{\text{T}}=\left(8.05\times \text{A}663+20.29\times \text{A}645\right)\times \text{v}/\left(1000\times \text{m}\right)$$

where *W*_*a*_, *W*_*b*_, and *W*_*T*_ represent the concentrations (mg L^−1^) of chlorophyll a, chlorophyll b, and total chlorophyll, respectively; A645 and A663 denote the absorbance values at 645 nm and 663 nm, respectively; *m* stands for the weight of leaf sample (g); and *v* represents the volume of extraction solution (mL).

#### Determination of antioxidant enzyme activity and malondialdehyde

The activities of peroxidase (POD), superoxide dismutase (SOD), catalase (CAT), total antioxidant capacity (T-AOC), and malondialdehyde (MDA) in *S. trifasciata* were determined using commercially available kits: E-BC-K227-S and E-BC-K019 were procured from Elabscience Biotechnology Co., Ltd (Wuhan, China), while A007-1–1, A015-1–2, and A003-1–1 were acquired from Nanjing Jiancheng Institute of Bioengineering (Nanjing, China). The plant samples, both roots and leaves, were initially cut into pieces. Pre-cooled saline was added, and homogenization was achieved under ice water bath conditions. The resulting mixture was then centrifuged for 15 min at 4 ℃, 4000 rpm, utilizing a high-speed refrigerated centrifuge (HC-2518R, Anhui, China). The supernatant extracted was used for kit-based detection following the manufacturers’ instructions. Absorbance readings were obtained using a UV–VIS spectrophotometer (UV-5200, METASH, Shanghai, China) at specific wavelengths: 550 nm for SOD, 405 nm for CAT, 420 nm for POD, 520 nm for T-AOC, and 532 nm for MDA. Each measurement was repeated three times for statistical reliability.

#### Determination of phytohormone content

The levels of phytohormone, including indole-3-acetic acid (IAA), ethylene (ETH), gibberellin (GAs), cytokinin (CTK), and abscisic acid (ABA), were quantified using kits obtained from Shanghai mlbio Co., LTD. (Shanghai, China).

#### High-throughput determination of endophytic flora

Root and leaf samples of *S. trifasciata* were collected and subsequently submitted to Sangon Biotech Co., Ltd (Shanghai, China) for high-throughput sequencing to examine the diversity of endophytic microbial communities within the samples. The 16S sequencing of endophytes in both roots and leaves was carried out using universal primers 341F (5′—CCTACGGGNGGCWGCAG -3′) and 805R (5′—GACTACHVGGGTATCTAATCC -3′).

### Data analysis

Analysis of variance (ANOVA) was employed for assessing the formaldehyde removal ability of *S. trifasciata*, and the variations in different indices.

## Results and discussion

### The formaldehyde removal ability

The system parameters in the test chamber were assessed at an inlet formaldehyde concentration of 5 ± 0.5 ppm. The recovery rate of formaldehyde consistently fell between 90.00 and 99.99% within the initial 6 h, with an average recovery rate of 98.76% over a 24-h period. Throughout the experiment, no significant differences were observed between the control group and the blank group, aligning with the data observed for potted *S. trifasciata* before formaldehyde fumigation (day 0). Therefore, the changes in various indicators during the experimental process (days 1–7) were directly compared with those on day 0.

**Fig. 2:**
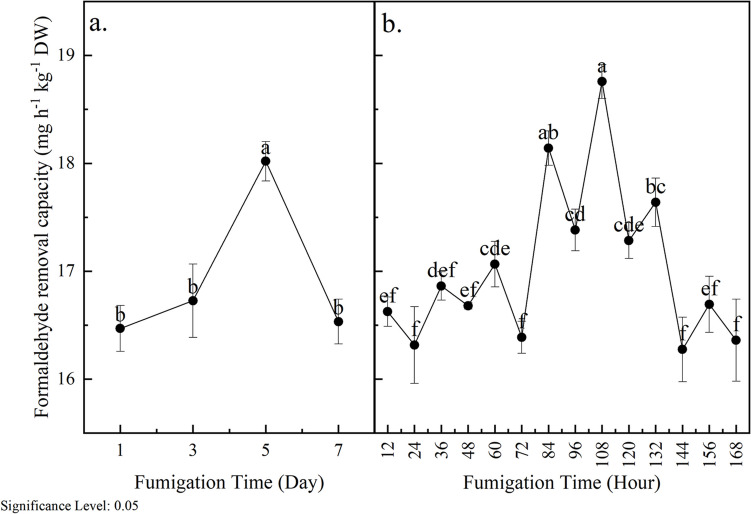
Formaldehyde removal capability of *S. trifasciata* over time with fumigation ((**a**) variations in average daily formaldehyde removal capacity; (**b**) fluctuations of formaldehyde removal capacity during day and night)

The formaldehyde removal capacity of potted *S. trifasciata* plants exposed to 5 ppm formaldehyde gas is depicted in Fig. 2a. During the 7-day dynamic fumigation period, the average formaldehyde removal rate of S. *trifasciata* was recorded at 16.93 mg h^−1^ kg^−1^ DW. From the 1st to the 5th day, the formaldehyde removal rate gradually increased, peaking at 18.02 mg h^−1^ kg^−1^ DW. However, from the 5th to the 7th day, the removal rate exhibited a decline, reaching 16.53 mg h^−1^ kg^−1^ DW on the 7th day. Over the 7-day fumigation period (Fig. 2b), the removal capacity of *S. trifasciata* for formaldehyde was higher during the day (odd-numbered 12 h) compared to nighttime (even-numbered 12 h). Throughout these 7 days, the average formaldehyde removal rate of *S. trifasciata* during the daytime (17.31 mg h^−1^ kg^−1^ DW) was significantly higher than that at night (16.56 mg h^−1^ kg^−1^ DW). This finding aligns with previous studies reported by Li et al. (2019). Meanwhile, Zuo et al. (2023) reported that CO_2_ concentration had no significant effect on the formaldehyde removal ability and physiological characteristics of plants. However, they found that light intensity positively influenced the formaldehyde removal rate of *S. trifasciata*. The variation in light intensity corresponded to changes in the formaldehyde removal capacity of *S. trifasciata* during the daytime and nighttime, indicating that light is a limiting factor in the plant’s ability to remove pollutants like formaldehyde.

This observed change may be attributed to the inherent life activities of *S. trifasciata*, because the absorption and degradation of atmospheric pollutants depend on the metabolic activities of plants (Khalifa et al. 2023). For example, Khaksar et al. (Khaksar et al. 2016a, b, 2017) found that under formaldehyde stress, the increase of IAA level could reduce the intracellular reactive oxygen species (ROS) level, improve the formaldehyde removal ability of *E. milii* plants, and protect them from formaldehyde stress. At the same time, endophytes with plant growth-promoting properties can induce the growth and development of plants under formaldehyde stress, and enhance the repair ability of their hosts (Khaksar et al. 2016c). In addition, antioxidant enzymes such as POD, SOD, and CAT are essential in clearing excess ROS and maintaining oxidative balance, thereby resisting the damage of formaldehyde to plants (Li et al. 2019).

#### The variations in chlorophyll levels

**Fig. 3:**
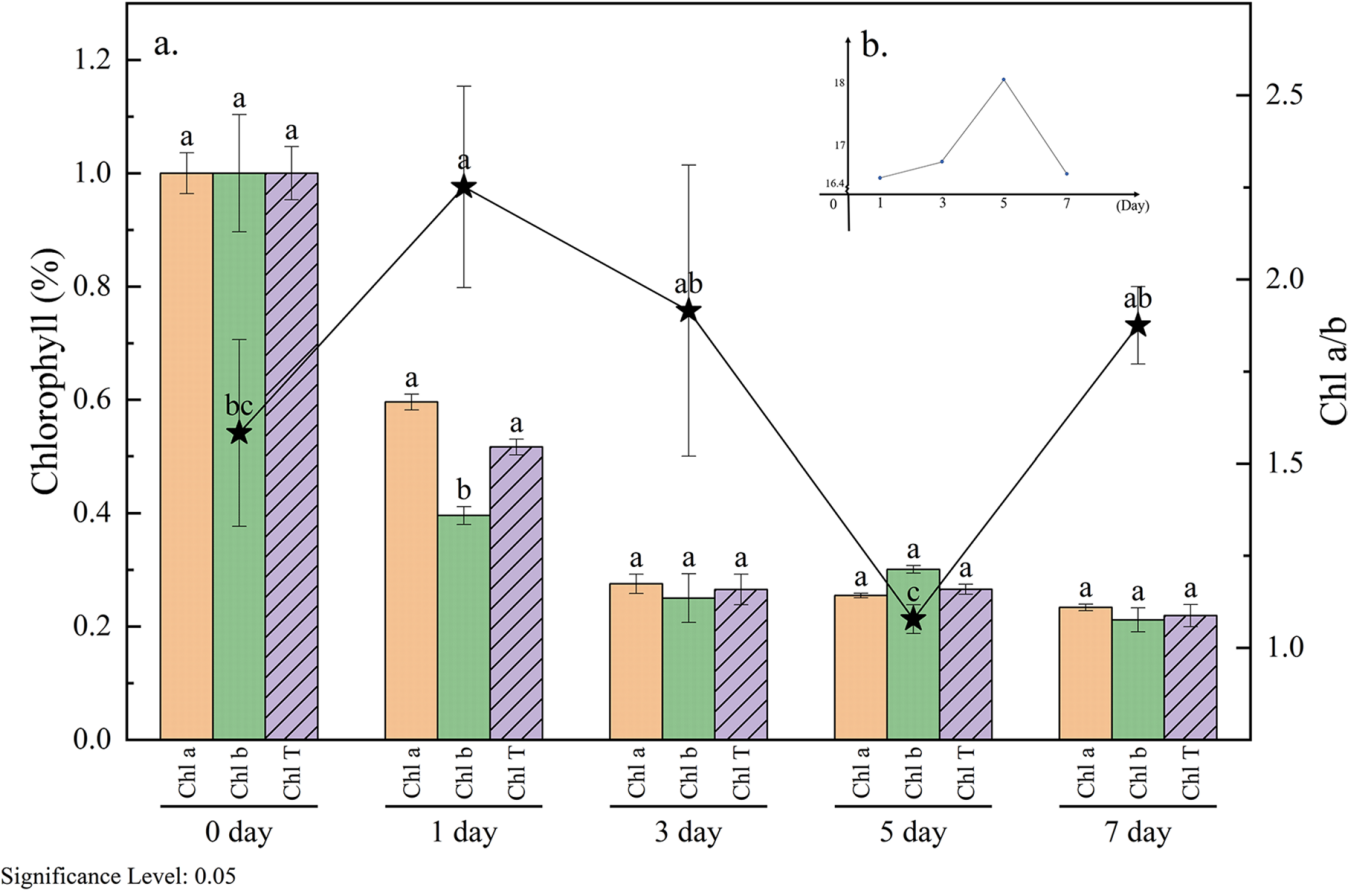
Changes in chlorophyll content of *S. trifasciata* with fumigation time ((**a**) changes in chlorophyll content; (**b**) changes of formaldehyde removal ability, see Fig. 2a for details)

Chlorophyll serves as a crucial pigment in plant photosynthesis, with its content reflecting the photosynthetic capacity of plants. Upon exposure to formaldehyde at a concentration of 5 ppm, the chlorophyll a, chlorophyll b, and total chlorophyll contents in *S. trifasciata* leaves exhibited a gradual decline (Fig. 3). This downward trend persisted until stabilization after the 3rd day, with all values decreasing by approximately 80% compared to pre-fumigation levels by the 7th day. In a similar vein, Li et al. (2019) reported a study wherein exposure to a 1-ppm formaldehyde concentration for 7 days led to a chlorophyll content reduction in *C. comosum* plants ranging from 6.95 to 25.32%. Additionally, a comprehensive review has suggested that chlorophyll content tends to decrease under higher stress levels (Agathokleous et al. 2020).These changes indicated that formaldehyde stressed *S. trifasciata* and affected its photosynthesis, which in turn will impact plant growth and subsequent recovery.

Nevertheless, the chlorophyll a/chlorophyll b ratio (Chl a/b ratio) in *S. trifasciata* exhibited higher values over the 7 days of fumigation compared to the pre-fumigation period, with the exception of the fifth day (Fig. 3). On the 1st day of fumigation, the Chl a/b ratio reached its peak at 2.26, while on the 5th day, it dropped to the lowest value of 1.09. Notably, this trend ran contrary to the changes observed in formaldehyde removal ability over the 7-day period.

Light harvesting complexes I (LHCI) capture light energy for Photosystem I (PSI), and light harvesting complexes II (LHCII) serve Photosystem II (PSII) to drive photosynthesis. The Chl a/b ratio is intricately linked to the PSI/PSII ratio and LHCII/PSII ratio (Schiphorst et al. 2022). Consequently, the Chl a/b ratio acts as a reflective measure of leaf photosynthetic activity intensity. The increased Chl a/b ratio represents a chloroplast’s adaptive defense mechanism in plant leaf cells against adverse environmental conditions (Loggini et al. 1999). On the 5th day, both chlorophyll a and b contents decreased, with a more pronounced decline in chlorophyll a content, ultimately resulting in a decrease in the Chl a/b ratio on the 5th day. This dynamic may contribute to the observed peak in the formaldehyde removal ability of *S. trifasciata* on the 5th day.

#### The variations in antioxidant oxidase activity levels

Elevated levels of pollutants (such as formaldehyde) can lead to a significant increase in ROS within plants, resulting in cell damage and impeding overall plant growth (Permana et al. 2023). To counteract the detrimental effects of ROS, plants employ antioxidant enzymes such as SOD, CAT, and POD (Khan et al. 2023b). Consequently, higher antioxidant enzyme activity correlates with increased ROS content.

**Fig. 4:**
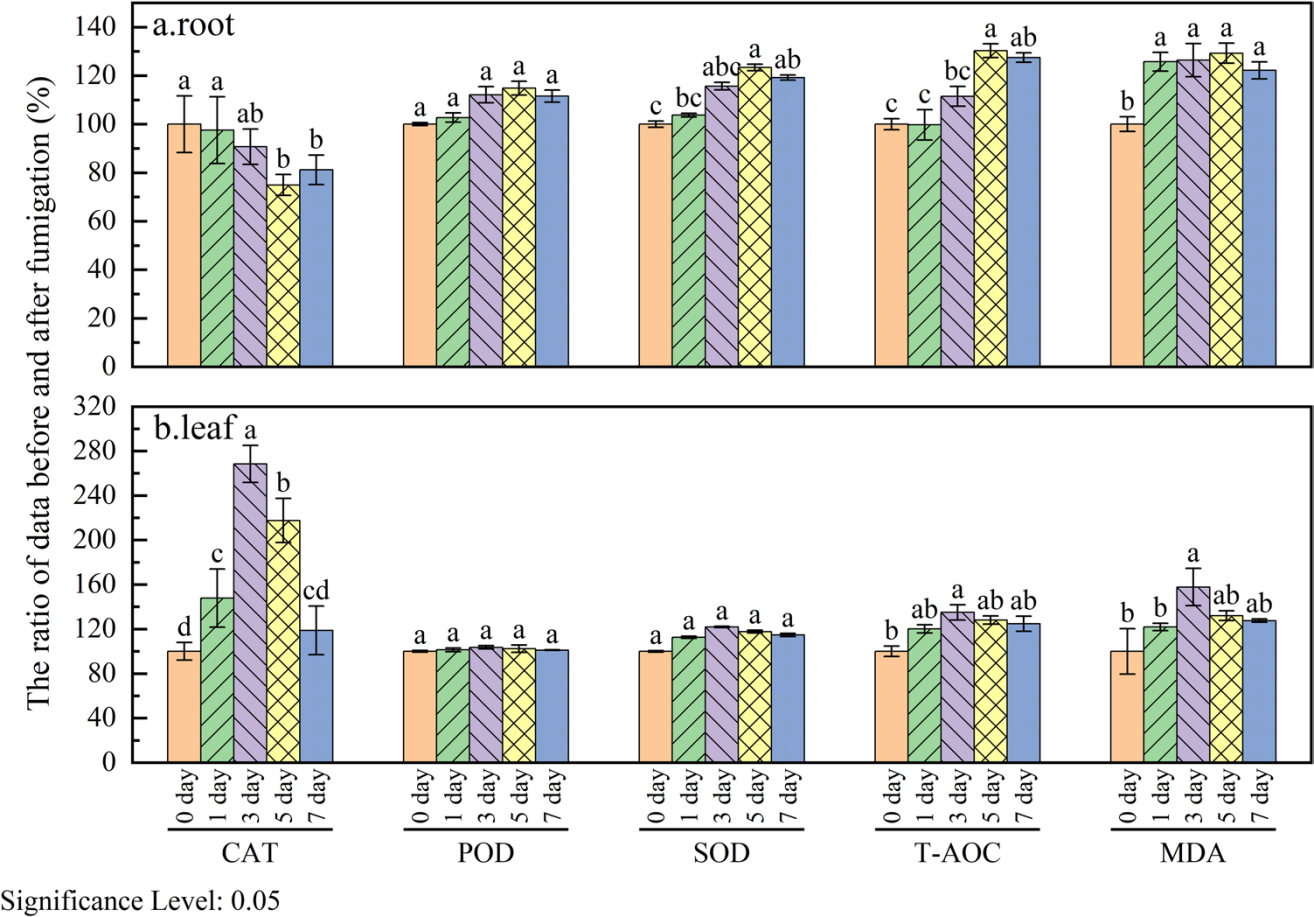
Changes of antioxidant enzyme activity in roots and leaves of *S. trifasciata* with fumigation time ((**a**) changes of antioxidant enzyme activity in roots; **b** changes of antioxidant enzyme activity in leaves)

The enzymatic antioxidant system played a crucial role in mitigating the damage induced by ROS accumulation in *S. trifasciata*. During fumigation, fluctuations in CAT and POD activities in the roots of *S. trifasciata* were observed (Fig. 4a). From the 1st to the 5th day, CAT activity significantly decreased, concomitant with an increase in POD activity. By the 5th day, CAT and POD activities reached 74.95% and 114.84%, respectively, of their pre-fumigation levels. Subsequently, from the 5th to the 7th day, CAT activity rebounded to 81.20%, while POD activity slightly decreased to 111.59% of pre-fumigation levels. The dynamic changes in SOD activity in the roots were consistent with those of POD. SOD activity rose to 122.07% of pre-fumigation levels within the initial 1st to 5th day, followed by a subsequent decline. This pattern may be attributed to the intense formaldehyde stress during the first 5 days, triggering a surge in ROS production (Khan et al. 2023b). In response, SOD synthesis increased, reaching its peak on the 5th day.

During the 1st to 5th day of fumigation, CAT activity decreased while POD activity increased, suggesting that under formaldehyde stress, most of H_2_O_2_ in *S. trifasciata* roots was reduced to H_2_O through POD catalysis, that is, POD played a major role. From the 5th to the 7th day of fumigation, CAT activity increased, while POD and SOD activities decreased. This shift may signify effective stress control in *S. trifasciata* roots, leading to a return of antioxidant enzyme activities to pre-fumigation levels. Consequently, *S. trifasciata* exhibited its highest formaldehyde removal ability on the 5th day.

In addition to antioxidant enzymes, other antioxidant substances contribute to the intricate antioxidant mechanisms in plants. The comprehensive antioxidant capacity of plants, encompassing both enzymatic and non-enzymatic oxidation systems, is encapsulated by T-AOC. On the subsequent 1st to 5th days, the T-AOC in *S. trifasciata* roots exhibited a steady increase, peaking at 130.29% of pre-fumigation levels on the 5th day before showing a marginal decline. By the 7th day, T-AOC was sustained at 127.45% of pre-fumigation levels. The fluctuation in T-AOC was the same as the patterns observed in POD and SOD activities. The consistently elevated antioxidant capacity of *S. trifasciata* during fumigation maintained the production and transformation of ROS in a relatively stable state. This stability effectively mitigated ROS-induced damage to the plant, alleviating formaldehyde stress, promoting normal physiological activities, and enhancing the plant’s ability to remove formaldehyde. From the 5th to the 7th day, as ROS was effectively controlled, T-AOC slightly decreased, but its decline was smaller than that of POD and SOD. This suggests the involvement of other antioxidant substances in alleviating formaldehyde stress, such as total phenol, total flavone, proline, and total carotenoid (Khan et al. 2023b). The collective action of these antioxidant components underscores the plant’s robust defense against formaldehyde-induced oxidative stress.

The ROS produced by plants under stress can cause membrane lipids to undergo peroxidation, resulting in the production of malondialdehyde (MDA), which is a widely utilized indicator for assessing plasma membrane damage and reflecting plant responses to stress (Zhou et al. 2022). In Fig. 4, the MDA content in the roots of *S. trifasciata* consistently exceeded pre-fumigation levels during the fumigation period, reaching its peak on the 5th day at 129.25% of the baseline. From the 5th to the 7th day, MDA content decreased slightly and stabilized at 122.19% of pre-fumigation levels by the 7th day, maintaining an overall stable fluctuation range. This persistent elevation in MDA content in the first 5 days is attributed to the impact of formaldehyde stress, which caused a large number of ROS production and induced peroxidation of plant membrane lipid. The sustained high levels of MDA underscore the continual effect of formaldehyde stress on impeding normal growth and physiological activities in *S. trifasciata*, accompanied by damage to the lipid structure in its tissues. The subsequent decrease in MDA content from the 5th to the 7th day indicates a mitigation of the damage to membrane lipids in the tissue. This decrease was consistent with the decrease in POD, SOD, and T-AOC activity or content on days 5 to 7, related to their control of ROS. It not only alleviates the formaldehyde stress of *S. trifasciata*, enhances the plant’s tolerance to formaldehyde, but also improves its ability to remove formaldehyde.

In contrast to the roots, the activities or contents of CAT, POD, SOD, T-AOC, and MDA in the leaves of *S. trifasciata* exhibited a similar pattern of change (Fig. 4b). They showed an increase on the 1st to 3rd days of fumigation and subsequently decreased. The maximum values for CAT, POD, SOD, T-AOC, and MDA were reached on the 3rd day, recording at 268.43%, 103.52%, 121.88%, 135.01%, and 157.75% before fumigation, respectively. In the initial phase of fumigation, formaldehyde stress led to an upsurge in ROS production, prompting an increase in SOD activity to catalyze the conversion of ROS into H_2_O_2_. Notably, the rise in CAT activity in leaves, as the enzyme catalyzing the decomposition of H_2_O_2_ into H_2_O and O_2_, was significantly higher than that of POD, exhibiting a stark contrast to the decrease in CAT activity in roots. This distinction highlights a differential response of *S. trifasciata* leaves to formaldehyde stress compared to the roots. That is, in the roots, POD mainly acts on H_2_O_2_, while in the leaves, CAT played a dominant role in directly decomposing H_2_O_2_ into H_2_O and O_2_ (Hegedüs et al. 2001). Additionally, the activities or contents of these five indicators in leaves peaked on the 3rd day, whereas in the roots, the turning point typically occurred on the 5th day. This suggests that the response of leaves to formaldehyde stress precedes that of the roots, possibly due to the direct exposure of leaves to formaldehyde gas. The MDA content in leaves reached the highest earlier than that in roots, indicating that lipid peroxidation occurred faster in leaves than in roots. To mitigate the adverse effects of formaldehyde on *S. trifasciata* leaves, the activities or contents of antioxidant enzymes and T-AOC increased accordingly. By the 3rd day of fumigation, since the stress on the leaves was controlled, so the five indicators of leaves all decreased on the 3rd to 7th day. However, the root indicators showed that the stress on the roots had not been effectively relieved after the 3rd day. Therefore, after the 3rd day, the root mainly played a role in alleviating the stress of formaldehyde on the plant. And rhizosphere degradation emerges as a crucial pathway when plants confront formaldehyde exposure (Khalifa et al. 2023), it can be inferred that the stress induced by formaldehyde on the plant was more effectively alleviated by the roots.

#### The variations in phytohormone levels

Gene expression, growth, development, and the responses of plants to specific environmental stimuli are regulated by phytohormone, so phytohormone play a crucial role in regulating plant defense responses to biological stress (Khan et al. 2023a). The common plant hormones mainly include IAA, ETH, GAs, CTK, and ABA.

**Fig. 5:**
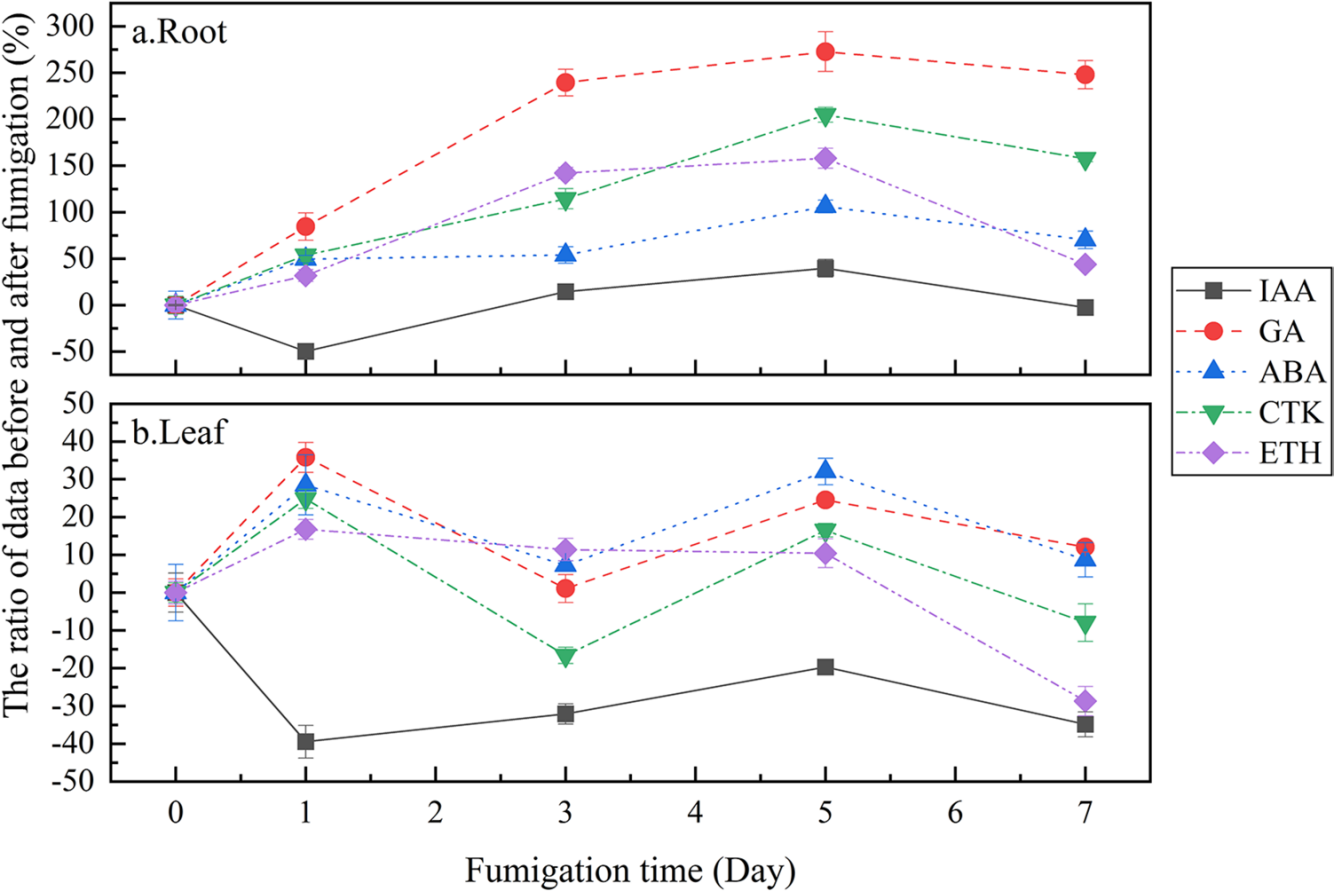
Changes of phytohormone content in roots and leaves of *S. trifasciata* with fumigation time ((**a**) changes of phytohormone content in roots; (**b**) changes of phytohormone content in leaves)

The experimental findings for the roots of the plants are illustrated in Fig. 5a. On the 1st day of fumigation, IAA content in the roots of *S. trifasciata* showed a brief decrease, decreasing by 47.62%. Subsequently, it exhibited a consistent increase, reaching its zenith on the 5th day at 146.01% compared to pre-fumigation levels. However, by the 7th day, the IAA content had declined and returned to the baseline. In contrast, the ETH content consistently surpassed pre-fumigation levels, reaching its peak on the 5th day at 257.98% before gradually decreasing to 143.93% on the 7th day. On the 1st day of fumigation, the decrease in IAA may be due to the physiological activity of plants being affected by formaldehyde stress, resulting in a reduction in IAA synthesis by root tips. The increase in IAA is recognized as beneficial for pollutant removal by plants since higher IAA levels restrict the accumulation of ROS (Khaksar et al. 2017). So, due to formaldehyde stress, the IAA content continued to rise, peaking on the 5th day., As one of the regulatory hormones, ETH exhibits an increased synthesis rate under the influence of biological and abiotic stress (Khan et al. 2023a). Therefore, during the 1st to 5th days, formaldehyde stress instigated an augmentation in endogenous ETH biosynthesis in the roots. However, it is noteworthy that as its content escalates, the inhibitory effect of ETH on plant growth also increases (Mayak et al. 2004). Thus, in the later stage of fumigation, after the formaldehyde stress is alleviated, ETH content decreased rapidly. Simultaneously, the negative feedback regulation of IAA by high concentrations of ETH (Khaksar et al. 2016a) contributed to a decrease in IAA content. Consequently, IAA also reverted to pre-fumigation levels on the 5th to 7th days.

In the leaves of *S. trifasciata* (Fig. 5b), the IAA content consistently remained lower than pre-fumigation levels. Although it increased from day 1 to 5 and reached its peak on the 5th day, it still 19.66% lower than pre-fumigation levels. Subsequently, on the 5th to 7th days of fumigation, the IAA content exhibited a decline, reaching 65.13% of pre-fumigation levels on the 7th day. On the 1st day of fumigation, the ETH content was at its highest, registering 116.74% before gradually decreasing but remaining higher for the first 5 days compared to pre-fumigation levels. On the 5th to the 7th day, it decreased rapidly to 71.28% of the pre-fumigation level. The response of *S. trifasciata* to formaldehyde stress prompted a slight increase in ETH content on the 1st days, aligning with findings by Stearns and Glick, who also observed elevated endogenous ETH biosynthesis under stress conditions (Stearns and Glick 2003). Moreover, the leaves were directly exposed to formaldehyde gas, formaldehyde stress caused ROS accumulation, which affected the life activities of leaves. Simultaneously, leaf tip is one of the main production sites of IAA, which led to the decrease of IAA content in leaves on the 1st day after exposure to formaldehyde. Under abiotic stress, plants typically generate IAA to mitigate stress and regulate ROS accumulation (Khaksar et al. 2016b). Consequently, the IAA content increased from the 1st to the 5th day, peaking correspondingly with the highest formaldehyde removal ability of *S. trifasciata* during the same period. The elevated IAA levels led to a rapid decline in ETH content on the 5th to 7th days, reaching a point 34.87% lower than pre-fumigation levels on the 7th day. This phenomenon is attributed to IAA’s control over ROS accumulation, limiting the activation of 1-aminocyclopropane-1-carboxylic acid (ACC), a precursor substance of ETH synthase, by ROS, thereby contributing to the observed decrease in ETH (Khaksar et al. 2016a). It is noteworthy that the IAA content in leaves during fumigation always remained lower than pre-fumigation levels. This discrepancy may arise from the fact that the main synthesis site for IAA is at the top of the bud, and the generated IAA is transported to the root through the vascular tissue (Rigas et al. 2013). This transport mechanism results in an increase of IAA in the roots, contributing to the observed disparity in IAA levels between leaves and roots.

During fumigation, the fluctuations in GAs, CTK, and ABA content were similar in different parts of *S. trifasciata* (both in the roots and leaves). In the roots of *S. trifasciata*, their content exceeded the pre-fumigation level, rising from day 1 to 5 and reaching the peak on the 5th day. Specifically, the contents of GAs, CTK, and ABA rose to 372.74%, 304.76%, and 206.12% before fumigation, respectively. Subsequently, the content of GAs and ABA decreased from the 5th to 7th day to 347.95% and 170.21% before fumigation, respectively. As recognized, ABA functions as a stress hormone, mitigating stress by regulating guard cells and inducing stomatal closure, and its accumulation helps to enhance plant stress resistance (Ma et al. 2018). Additionally, ABA can activate the expression of antioxidase-related genes, improve the activity of antioxidant enzymes such as SOD, consequently enhancing the ability to clear ROS and reducing ROS-induced cellular damage (Gomez-Cadenas et al. 2015). This also can explain the increased activity of antioxidant enzymes. Therefore, when confronted with formaldehyde stress, *S. trifasciata* responded by increasing ABA concentration. This elevation in ABA levels suggests a strategic adaptation to counteract the detrimental effects of stress on the plant, enhancing its overall resilience and antioxidative defense mechanisms.

However, as a plant hormone, ABA can induce leaf shedding and inhibit cell growth (Chen et al. 2020). To counterbalance the adverse effects of increased ABA content on plants and maintain hormone equilibrium, changes in GAs and CTK content were synchronized with ABA. Because GAs and CTK themselves can regulate plant growth, and in addition to the antagonistic relationship between ABA and GAs (Vishal and Kumar 2018), there is also antagonism between CTK and ABA in regulating stomata, which can enhance plant stress resistance by regulating stomatal opening and maintaining hormone balance (Zhou et al. 2022). During the initial 1st to 5th days of stress, the content of GAs in the roots significantly increased. This finding is in line with a similar study by Kittipornkul et al. (Kittipornkul et al. 2023), where the content of gibberellin in *S. trifasciata* also showed a significant increase in a water shortage environment. Notably, both auxin and GAs can promote plant growth, but auxin can also promote the accumulation of biologically active GAs (Weston et al. 2009). Therefore, the observed increase in IAA content from the 1st to the 5th day may be another contributing factor to the rise in GA content in the roots of *S. trifasciata*.

The peak values of GAs, CTK, and ABA content in the leaves appeared on the 1st day, which were 135.79%, 124.81%, and 128.50% compared to the levels before fumigation, respectively. Subsequently, these hormone levels decreased to their lowest points on the 3rd day, with values 1.07%, − 16.62%, and 7.26% higher than before fumigation, respectively. However, they exhibited an upward trend again from the 3rd to the 5th day before decreasing within the 5th to 7th day. In addition to its antagonistic role against the stress hormone ABA, CTK also has a role in inhibiting plant aging (Ullah et al. 2018), providing a mechanism to regulate the growth of stressed *S. trifasciata*. Consequently, the increase in GAs, CTK, and ABA content in the leaves on the 1st day of fumigation aimed to sustain the normal growth of leaves directly exposed to formaldehyde. Notably, CTK and ABA are usually produced in plant roots (He et al. 2012), so, the CTK and ABA detected in the leaves of *S. trifasciata* mainly originated from root transport. During the initial 1st to 3rd days of fumigation, a substantial portion of the CTK and ABA produced by the roots likely remained in the roots to alleviate stress in the roots of *S. trifasciata*. This is further supported by the rapid increase in the content of CTK and ABA observed in the roots in the 1st to 3rd days. Consequently, the limited transport of CTK and ABA to the leaves during this period resulted in a decline in their content in the leaves from the 1st to the 3rd day. With the gradual relief of root stress, more CTK and ABA were transported to the leaves. Therefore, on the 3rd to the 5th day, the content of CTK and ABA in the leaves increased, contributing to the alleviation of leaf stress and the enhancement of leaf stress resistance.

By the 5th day, the stress on *S. trifasciata* had been effectively alleviated, accompanied by an enhancement in its formaldehyde removal ability. With the effective regulation of plant growth status and physiological activities, various hormones returned to normal levels before fumigation, achieving dynamic balance. Therefore, on the 5th to 7th day, all hormones in roots and leaves showed a downward trend. This dynamic interaction suggests a complex and interconnected hormonal regulation system within the plant to cope with stress and maintain hormonal homeostasis. The observed fluctuations in hormone levels over the course of the fumigation period highlight the dynamic and complex adaptive mechanisms of plant regulatory processes, shedding light on its ability to navigate environmental challenges and restore equilibrium.

**Fig. 6:**
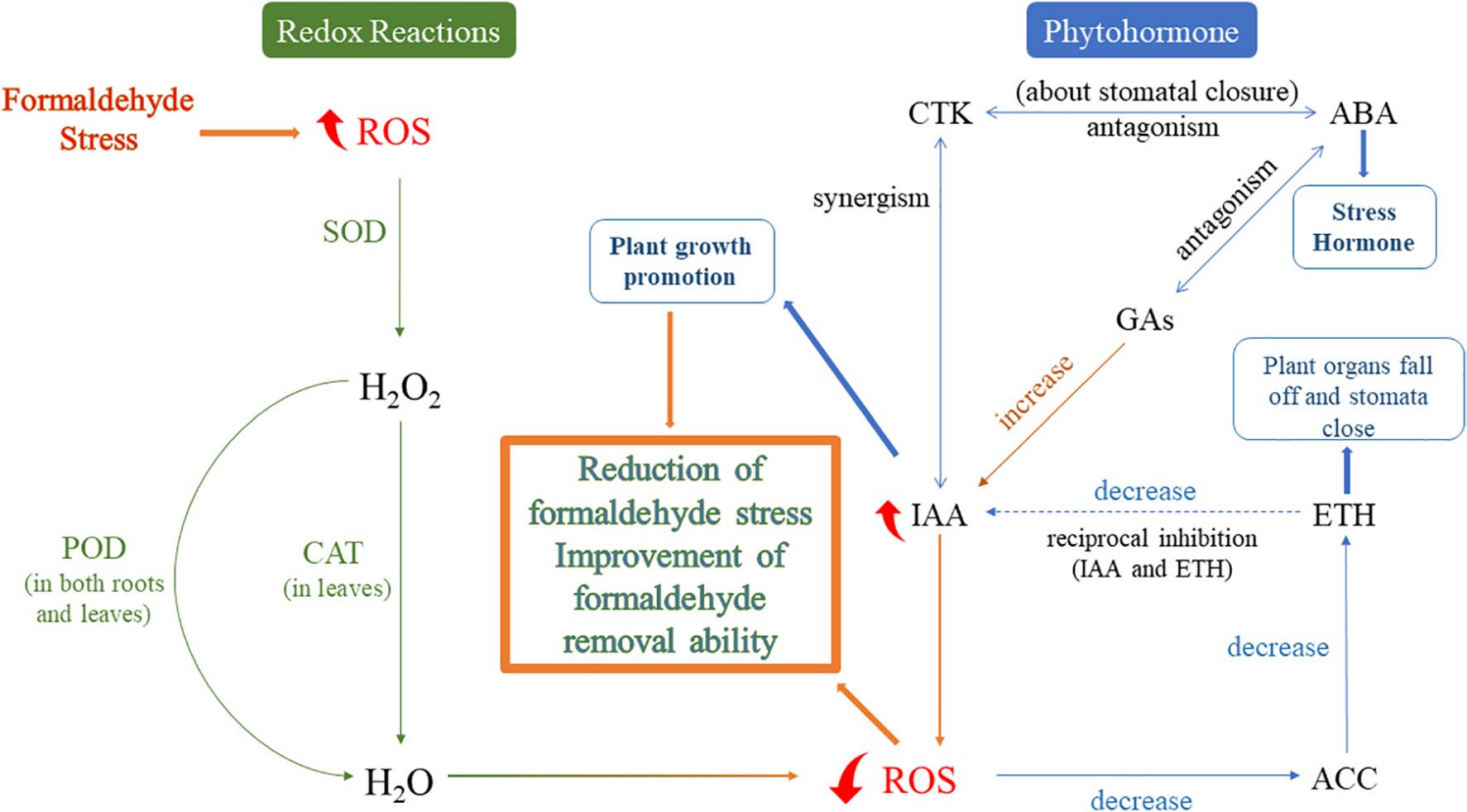
Mechanism of *S. trifasciata* response to formaldehyde stress

In general, the stress response of *S. trifasciata* under formaldehyde conditions is outlined in Fig. 6. Figure 6 provides a detailed account of specific physiological activities within the plant, such as Redox reactions and Phytohormone fluctuations, elucidating, for the first time, the dynamic changes in physiological characteristics of the plant under formaldehyde-induced conditions. This perspective offers novel insights into the mechanisms by which the plant degrades formaldehyde, contributing to our understanding of the dynamic physiological responses in plant systems.

#### The variations in relative abundance of endophytic flora

**Fig. 7:**
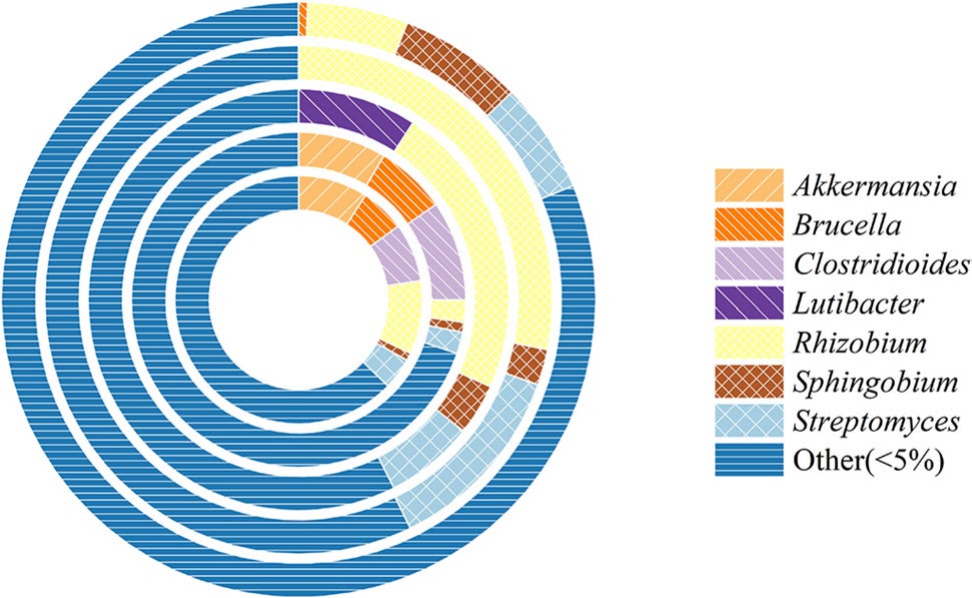
Relative abundance changes of endophytic flora in roots of *S. trifasciata*

**Fig. 8:**
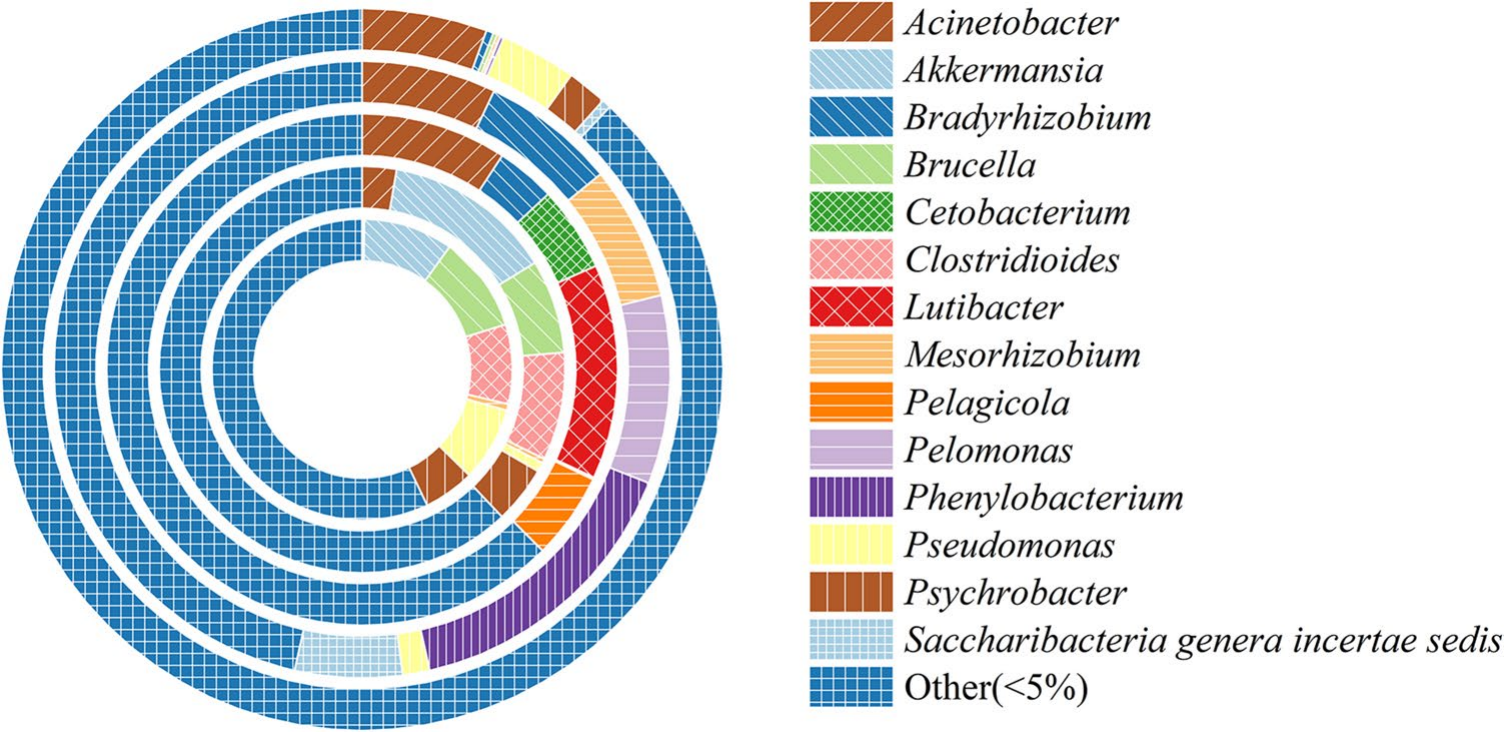
Relative abundance changes of endophytic flora in leaves of *S. trifasciata*

Endophytic microorganisms, residing within plant tissues, play a pivotal role in providing various benefits to host plants, including enhanced nutrient availability, synthesis of plant growth hormones, and regulation of soil pathogens, thereby promoting overall plant growth (Khaksar et al. 2016a). The relative abundances of these endophytes during fumigation were found to be closely intertwined with the changes in physiological activities and the formaldehyde removal ability of *S. trifasciata*. The relative abundances of endophytic flora in both the roots and leaves of *S. trifasciata* under formaldehyde stress were determined, as illustrated in Fig. 7 and Fig. 8. The outcomes of our testing on day 3 and day 5 revealed statistically significant differences (*p* < 0.05) when contrasted with measurements taken on day 0. This investigation sheds light on the intricate relationship between endophytic microorganisms and the response of *S. trifasciata* to formaldehyde exposure. The following sections will delve into the specific findings and implications of these microbial interactions in the context of plant physiology and formaldehyde removal.

To delve into the core endophytic bacteria inhabiting the roots and leaves of *S. trifasciata*, microorganisms with a relative abundance surpassing 5% in the samples were identified as the core endophytic bacteria. These include *Akkermansia* sp., *Brucella* sp., *Clostridioides* sp., *Lutibacter* sp., *Rhizobium* sp., *Sphingobium* sp., and *Streptomyces* sp. Among these, *Akkermansia* sp., *Brucella* sp., and *Clostridioides* sp. are recognized for their potential to cause diseases in animals or humans. *Lutibacter* sp. is commonly associated with oceanic environments. Among the identified microorganisms, *Rhizobium* sp., *Sphingobium* sp., and *Streptomyces* sp. are notably relevant to plant growth as plant growth-promoting bacteria (PGPB). Their relative abundances exhibited an increase during fumigation. *Rhizobium* sp., known for its association with root nodules, demonstrates the ability to fix atmospheric N_2_ into ammonia, enhancing plant nutrient absorption, especially in legumes (Sessitsch et al. 2002; Aguilar et al. 2018). *Sphingobium* sp. and *Streptomyces* sp. contribute to root growth regulation and antioxidant enzyme system enhancement by stimulating the production of phytohormone, such as IAA, and facilitating the production of iron ions, dissolved phosphate, and nitrogen fixation (Olanrewaju and Babalola 2019; Jou et al. 2022). These activities promote plant growth and alleviate stress. This insight into the core endophytic bacteria further underscores the intricate interplay between microbial communities and the response of *S. trifasciata* to formaldehyde exposure. Subsequent sections will provide a comprehensive analysis of these findings and their implications in the broader context of plant physiology and formaldehyde removal.

The core endophytes identified in the leaves encompassed *Acinetobacter* sp., *Akkermansia* sp., *Bradyrhizobium* sp., *Brucella* sp., *Cetobacterium* sp., *Clostridioides* sp., *Lutibacter* sp., *Mesorhizobium* sp., *Pelagicola* sp., *Pelomonas* sp., *Phenylobacterium* sp., *Pseudomonas* sp., *Psychrobacter* sp., and *Saccharibacteria genera incertae sedis* sp. However, akin to the roots, only a limited number of microorganisms associated with plant growth—specifically *Acinetobacter* sp., *Bradyrhizobium* sp., *Mesorhizobium* sp., and *Pseudomonas* sp.—were identified, and they all belong to the category of PGPB. This selection of core endophytes in the leaves further emphasizes the crucial role of specific microbial communities in contributing to plant growth and stress mitigation.

The *Pseudomonas* sp. only had a relative abundance greater than 5% on the 0th day of fumigation, while *Bradyrizobium* sp. and *Mesorhizobium* sp. only on the 5th day. But the abundance of *Acinetobacter* sp. significantly increased throughout the entire fumigation period. *Acinetobacter* sp. is known to enhance biomass production by actively participating in the synthesis of phytohormone, such as IAA, and contributing to the formation of ferriferous carriers and extracellular polysaccharides (EPS). These activities lead to increased chlorophyll levels, reduced ROS production, diminished stress levels, and overall promotion of plant growth (Das and Sarkar 2018). Moreover, *Acinetobacter* sp. itself possesses formaldehyde-resistant characteristics. When introduced into *E. milii*, this endophytic bacterium significantly enhances the plant’s efficiency in removing gaseous formaldehyde (Khaksar et al. 2016a). The presence and dynamics of these specific endophytes contribute to the overall resilience and formaldehyde removal ability of *S. trifasciata*. The outcomes of our testing on day 3 and day 5 revealed statistically significant differences (*p* < 0.05) when contrasted with measurements taken on day 0.

Therefore, the observed rise in the relative abundance of *Rhizobium* sp., *Sphingobium* sp., *Streptomyces* sp., and *Acinetobacter* sp. likely contributes to the production of phytohormone and iron carriers, promoting nitrogen fixation and phosphate dissolution. This further aids *S. trifasciata* in reducing formaldehyde stress, bolstering its resilience to formaldehyde, and sustaining normal growth and life activities. In summary, the intricate interplay between endophytic microorganisms and the plant’s hormonal regulation mechanisms plays a pivotal role in fortifying the plant’s capacity to cope with environmental stressors such as formaldehyde exposure.

The fluctuations in other endophytic bacteria in leaves displayed a relatively irregular pattern, which could potentially account for the variability in phytohormone content in leaves. However, the underlying reasons for these irregular changes warrant further exploration and investigation.

## Conclusion

In this study, involving a dynamic formaldehyde fumigation experiment over 7 days, various physiological indicators including chlorophyll levels, phytohormone (IAA, ETH, GAs, ABA, CTK), antioxidant enzymes (CAT, POD, SOD), MDA, T-AOC, and the relative abundance of endophytic bacteria in both roots and leaves of *S. trifasciata* were analyzed.

The study revealed a decrease in chlorophyll content during fumigation, while the chlorophyll a/b ratio increased, indicating stress and compromised photosynthesis due to formaldehyde exposure. Throughout the 7-day fumigation, the formaldehyde removal ability of *S. trifasciata* exhibited a fluctuating pattern, reaching its peak on the 5th day at 18.02 mg h^−1^ kg^−1^ DW. This trend correlated with changes in phytohormone, POD, SOD, T-AOC, and MDA in the roots, the primary site of plant activities. These findings suggested that formaldehyde stress elevated ROS levels and lipid peroxidation in *S. trifasciata*, while phytohormone, antioxidant enzymes, and other substances increased to mitigate ROS damage, enhancing tolerance to formaldehyde stress and formaldehyde removal ability. As the plant adapted to the formaldehyde environment, these indicators trended towards normal levels before fumigation, indicating effective self-regulation and stress alleviation.

In the roots, an increase in the relative abundance of plant growth-promoting bacteria, capable of producing phytohormone, likely contributed to the observed changes in physiological indicators and formaldehyde removal ability during the fumigation process. Notably, CAT activity in the roots exhibited an opposite trend to that in the leaves, indicating distinct mechanisms of H_2_O_2_ conversion in response to stress. The turning points for physiological indicators in the leaves occurred on the 3rd day, preceding those in the roots, likely due to the direct exposure of leaves to formaldehyde gas.

This study provides valuable insights into the mechanisms underlying enhanced tolerance to formaldehyde stress and improved formaldehyde removal by *S. trifasciata*. The findings offer a theoretical foundation for future research on plant-mediated formaldehyde removal and pave the way for exploring synergistic approaches involving endophytic bacteria or genetic interventions to enhance treatment efficiency.

## Supplementary information

Below is the link to the electronic supplementary material.

Supplementary file1 (TIF 3240 KB)

Supplementary file2 (DOCX 642 KB)

## Data Availability

Data will be made available on reasonable request.
