# Supplementary figures and images for: Removal of formaldehyde from indoor air by potted Sansevieria trifasciata plants: dynamic influence of physiological traits on the process
Source: Environ Sci Pollut Res Int. 2024 Oct 29;31(54):62983–96. doi: 10.1007/s11356-024-35366-4 (PMC11599484; doi:10.1007/s11356-024-35366-4)

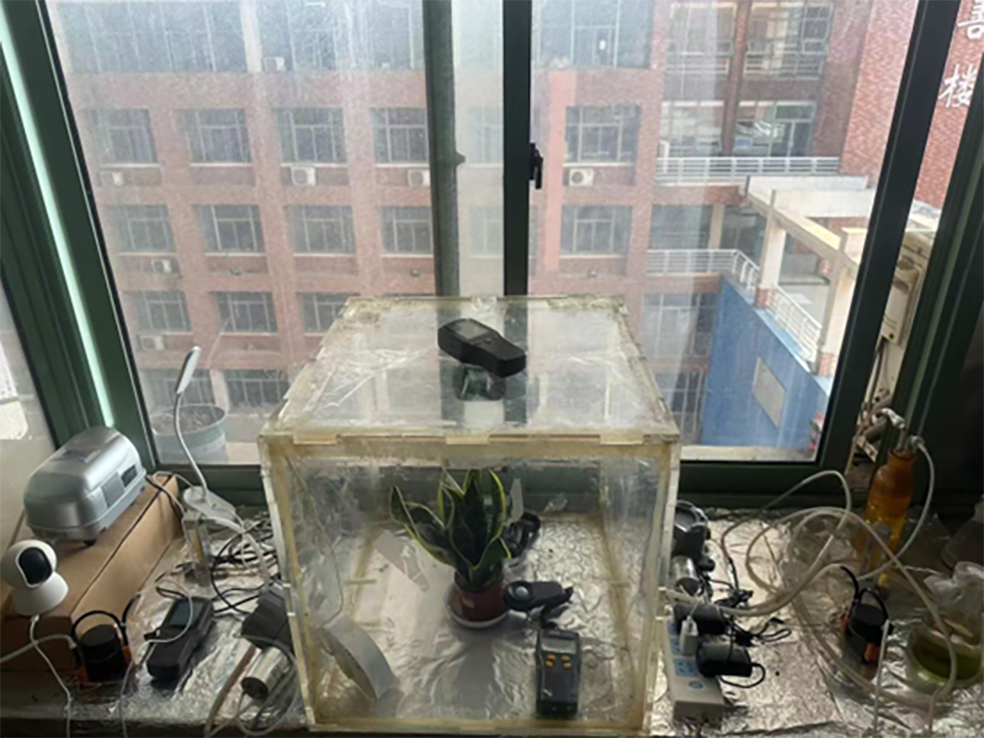

Supplement: Supplementary file 1 — Supplementary file1 (TIF 3240 KB) [file 11356_2024_35366_MOESM1_ESM.tif]
