# Supplementary material for: Removal of formaldehyde from indoor air by potted Sansevieria trifasciata plants: dynamic influence of physiological traits on the process
Source: Environ Sci Pollut Res Int. 2024 Oct 29;31(54):62983–96. doi: 10.1007/s11356-024-35366-4 (PMC11599484; doi:10.1007/s11356-024-35366-4)
Supplement: Supplementary file 2 — Supplementary file2 (DOCX 642 KB) [file 11356_2024_35366_MOESM2_ESM.docx]

**Supplementary material 2**

A leak test on the entire equipment was conducted before starting the experiments. The primary goal was to verify the airtightness of the fumigation system, and the secondary objective was to assess the extent of formaldehyde adsorption by the inner walls of the equipment. Before conducting the leak test, the average background formaldehyde concentration in the laboratory was measured over a 24-hour period, without operating the fumigation apparatus, and was determined to be 54.9 ± 9.95 µg/m³ (0.045 ± 0.0075 ppm). This concentration is significantly lower than the permissible exposure limit


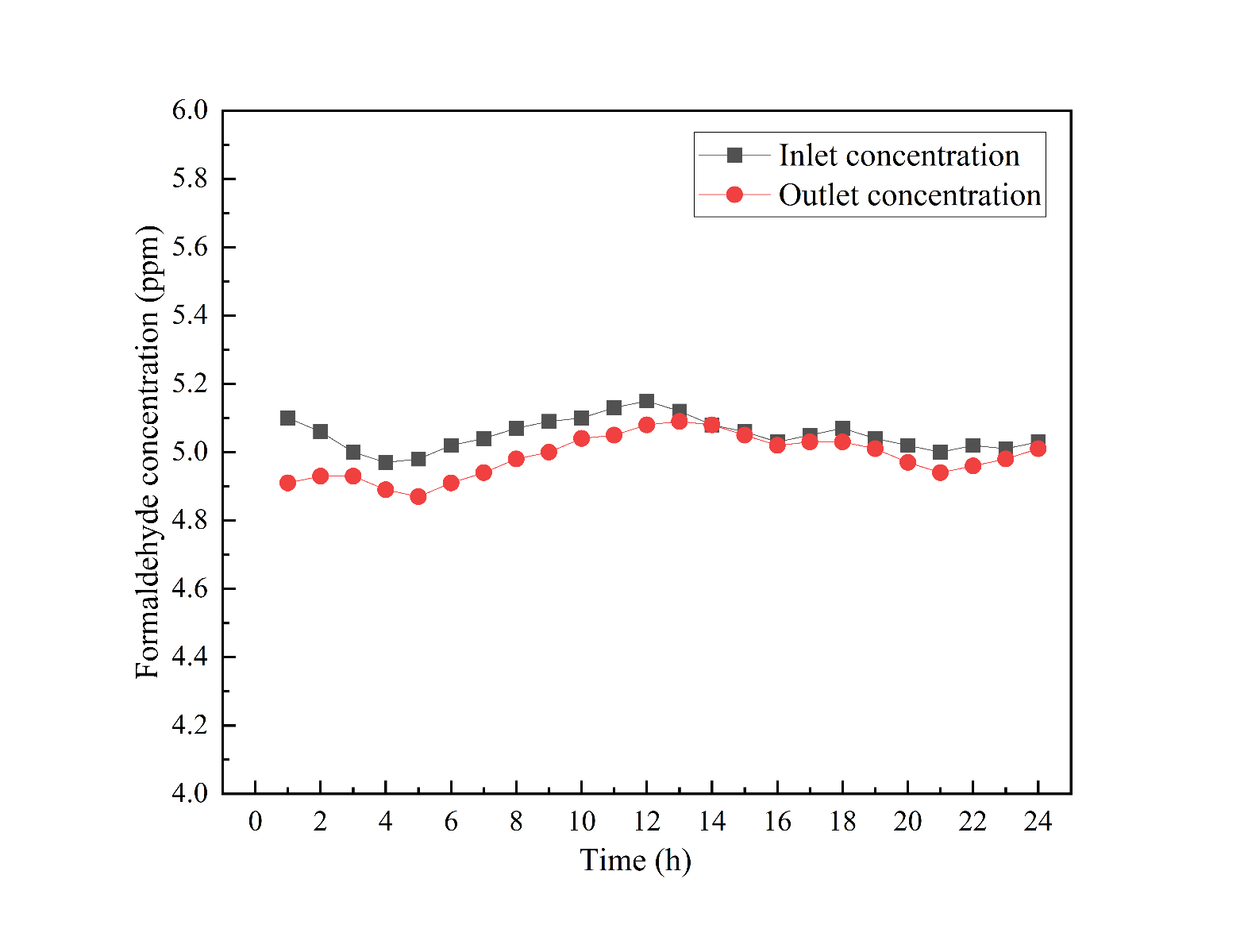


**Figure S1**. *Formaldehyde inlet and outlet concentration of experimental device*

(PEL) for formaldehyde in the workplace, which is 0.75 ppm, measured as an 8-hour time-weighted average (chrome-extension://efaidnbmnnnibpcajpcglclefindmkaj/https://www.osha.gov/sites/

default/files/publications/formaldehyde-factsheet.pdf). Then, for the leak test, formaldehyde gas with a concentration of 5 ± 0.5 ppm was continuously introduced into the experimental apparatus at a flow rate of 1.2 L/min for 24 hours. As is shown in Figure S1, during the 24-hour testing period, the inflow concentration of formaldehyde remained stable at 5.05±0.10 ppm, while the outflow concentration was maintained at 4.99±0.12 ppm. The system achieved an average gas recovery rate of 98.73%, demonstrating effective sealing and confirming its suitability for proceeding with further experiments.


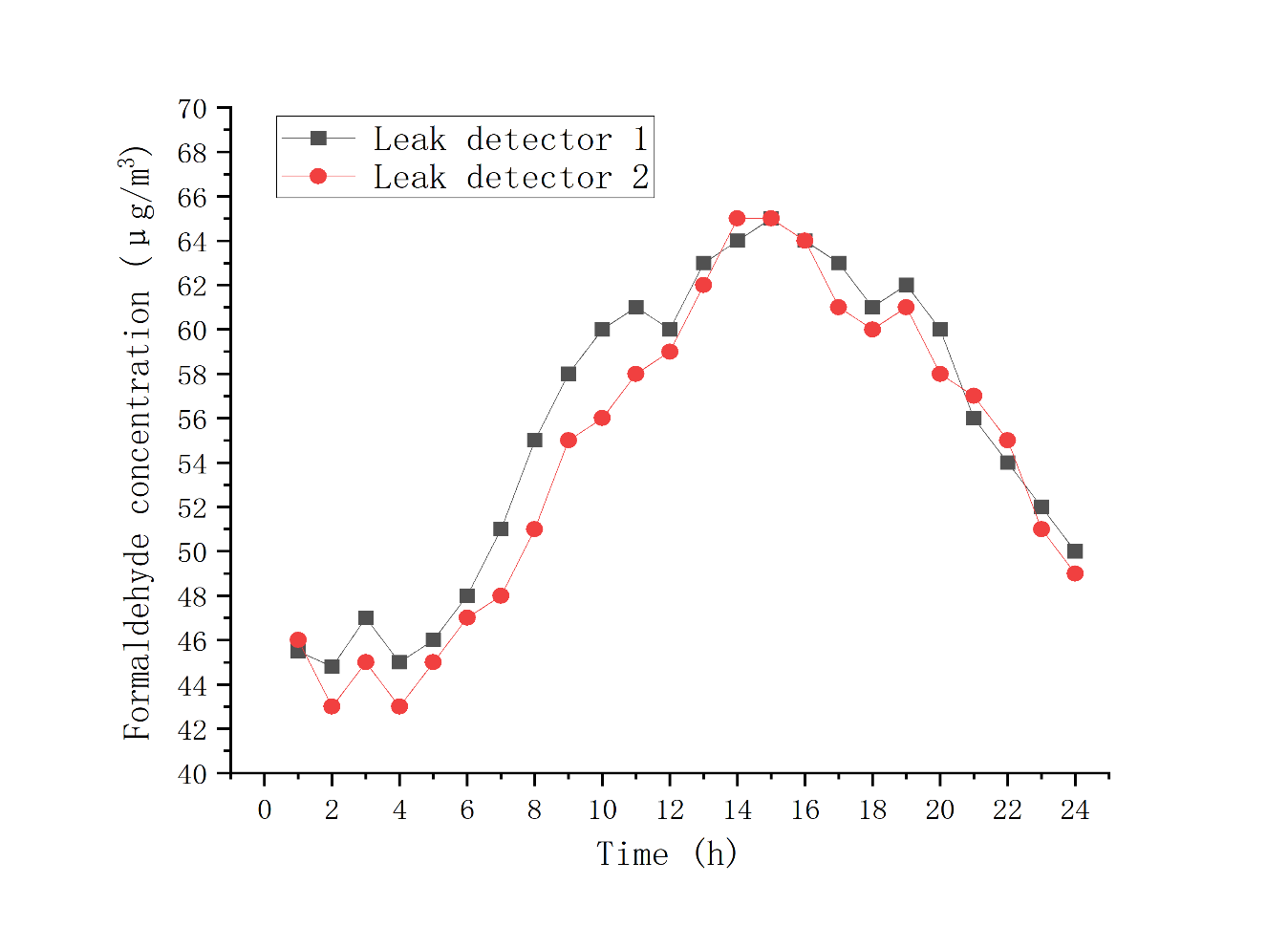
Figure S2 shows that the formaldehyde concentration around the apparatus ranges between 40 and 65μg/m³ (0.032 to 0.053 ppm), confirming that there is no leakage from the equipment to the

**Figure S2.** *Formaldehyde concentration around the fumigation apparatus*

laboratory environment, as the concentration has not exceeded the backgound levels. To further elaborate, the measured formaldehyde concentration levels surrounding the apparatus suggest effective containment and proper sealing of the equipment during operation. These results confirm that the fumigation system is functioning as intended, without any detectable leakage, thereby ensuring safety and compliance with health standards. Consistent monitoring of these levels is crucial to maintain a safe working environment.

Two types of devices, as shown in Figure S3, were utilised: one for leak detection (WP6912, AGERUISI, Beijing, China, Measurement Range/Resolution: 0.000-1.999 mg/m³ / 0.001 mg/m³) and the other for measuring formaldehyde concentrations during the fumigation process in the main experimental program (ADL-600B-HCHO, ANDEIL, Shenzhen, China, Measurement Range/Resolution: 0-10 ppm / 0.01 ppm). As is seen, the detection ranges and resolutions of these instruments differ significantly, ensuring accurate measurements associated with the specific requirements of each procedure.


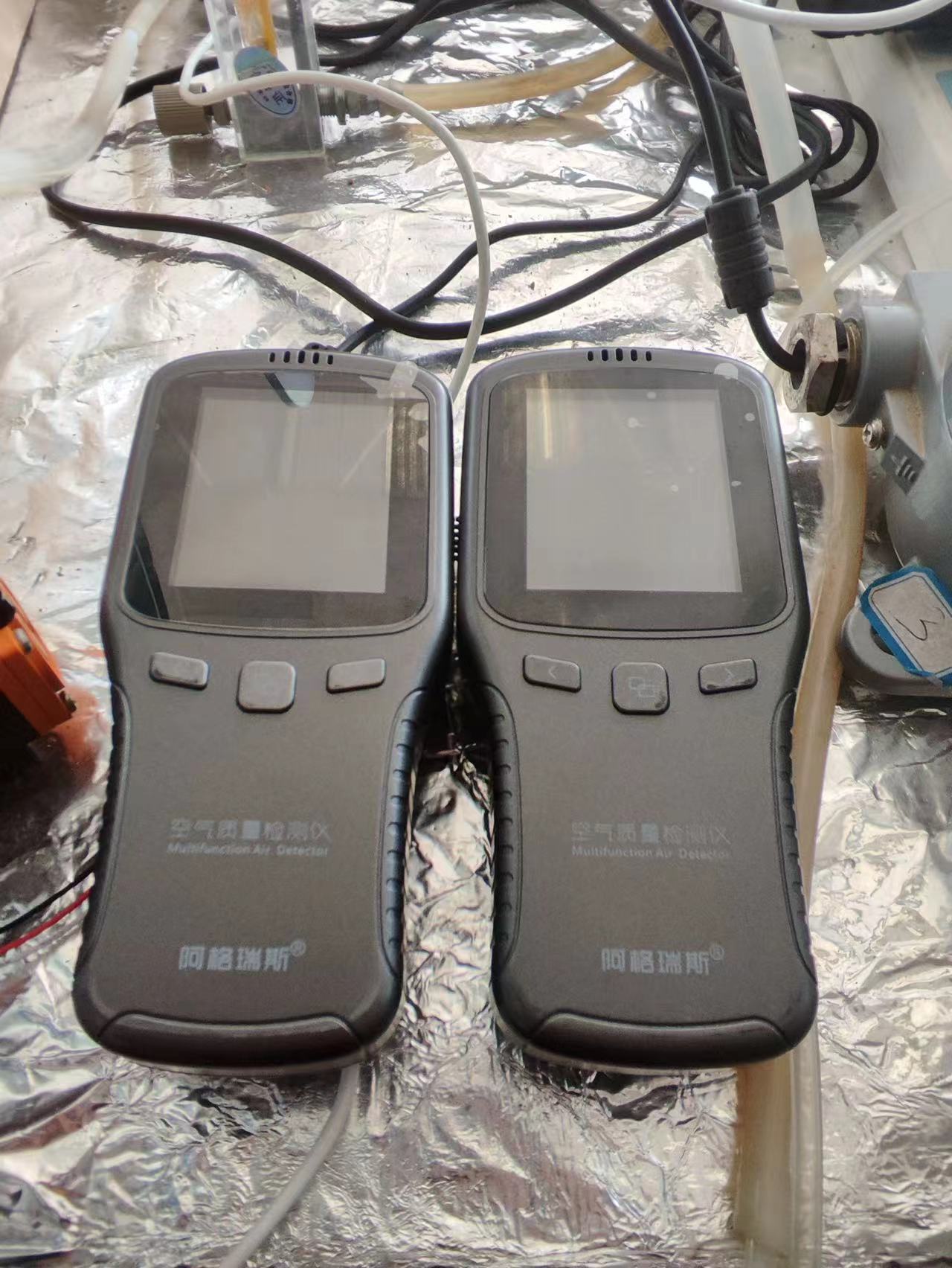

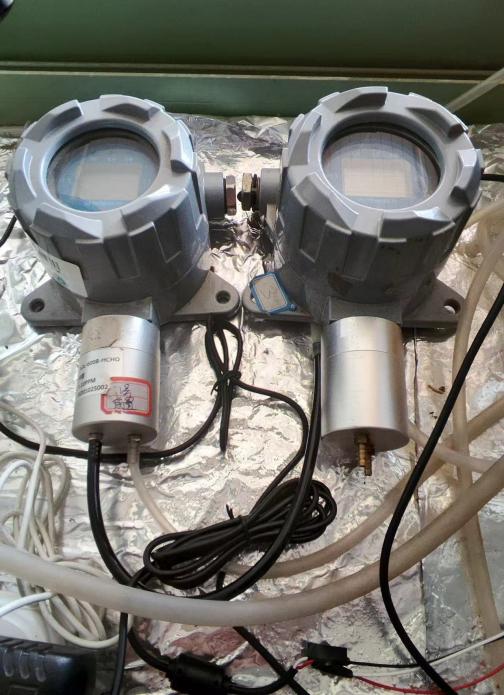


**2**

**1**

**Figure S3** Formaldehyde detectors used in the experiments. (*1) Leak detectors,WP6912, AGERUISI, Beijing, China, Measurement Range/Resolution: 0.000-1.999* *mg/m^3^ / 0.001mg/m^3^; (2) For the fumigation system,ADL-600B-HCHO, ANDEIL, Shenzhen, China, Measurement Range/Resolution: 0-10 ppm / 0.01ppm)*

The instruments used for the leak detection investigation were also continuously employed throughout the entire experimental procedure for safety purposes, ensuring timely detection of any potential formaldehyde leaks into the laboratory airspace.
